# Effects of Vedolizumab in Patients With Primary Sclerosing Cholangitis and Inflammatory Bowel Diseases

**DOI:** 10.1016/j.cgh.2019.05.013

**Published:** 2020-01

**Authors:** Kate D. Lynch, Roger W. Chapman, Satish Keshav, Aldo J. Montano-Loza, Andrew L. Mason, Andreas E. Kremer, Marcel Vetter, Manon de Krijger, Cyriel Y. Ponsioen, Palak Trivedi, Gideon Hirschfield, Christoph Schramm, Chung Heng Liu, Christopher L. Bowlus, Derek J. Estes, Daniel Pratt, Charlotte Hedin, Annika Bergquist, Annemarie C. de Vries, C. Janneke van der Woude, Lei Yu, David N. Assis, James Boyer, Henriette Ytting, Emina Hallibasic, Michael Trauner, Hanns-Ulrich Marschall, Luigi M. Daretti, Marco Marzioni, Kidist K. Yimam, Nicola Perin, Annarosa Floreani, Benedetta Terziroli Beretta-Piccoli, Jennifer K. Rogers, International Primary Sclerosing Cholangitis Study Group (IPSCSG), Cynthia Levy

**Affiliations:** ∗ Translational Gastroenterology Unit, Nuffield Department of Medicine, University of Oxford, Oxford, United Kingdom; ‡ Division of Gastroenterology, University of Alberta, Edmonton, Canada; § Department of Medicine 1, Friedrich-Alexander-University Erlangen-Nürnberg, Erlangen, Germany; ‖ Department of Gastroenterology and Hepatology, Amsterdam University Medical Centres, Location AMC, Amsterdam, The Netherlands; ¶ Centre for Liver and Gastroenterology Research, National Institute for Health Research, Birmingham Biomedical Research Centre, Birmingham, United Kingdom; # University Hospitals Birmingham, Birmingham, United Kingdom; ∗∗ Institute of Immunology and Immunotherapy, University of Birmingham, Birmingham, United Kingdom; ‡‡ Toronto Centre for Liver Disease, University Health Network and University of Toronto, Toronto, Canada; §§ First Department of Medicine and Martin Zeitz Centre for Rare Diseases, University Medical Center Hamburg-Eppendorf, Hamburg, Germany; ‖‖ Division of Gastroenterology and Hepatology, University of California Davis, Sacramento, California; ¶¶ Division of Hepatology, University of Miami, Miami, Florida; ## Autoimmune and Cholestatic Liver Center, Massachusetts General Hospital, Boston, Massachusetts; ∗∗∗ Patient Flow Gastrointestinal Diseases, Patient Area Gastroenterology, Dermatovenerology and Rheumatology, Karolinska University Hospital, Karolinska Institute, Stockholm, Sweden; ‡‡‡ Centre for Digestive Diseases, Division of Hepatology, Karolinska University Hospital, Karolinska Institute, Stockholm, Sweden; §§§ Department of Gastroenterology and Hepatology, Erasmus Medical Center, Rotterdam, The Netherlands; ‖‖‖ Liver Care & Transplantation Services, University of Washington Medical Center, Seattle, Washington; ¶¶¶ Yale Autoimmune and Cholestatic Liver Disease Program, Section of Digestive Diseases, Yale University School of Medicine, New Haven, Connecticut; ### Department of Hepatology, Rigshospitalet, University of Copenhagen, Copenhagen, Denmark; ∗∗∗∗ Division of Gastroenterology and Hepatology, Department of Internal Medicine III, Medical University if Vienna, Vienna, Austria; ‡‡‡‡ Department of Molecular and Clinical Medicine, Sahlgrenska Academy, University of Gothenburg, Gothenburg, Sweden; §§§§ Clinic of Gastroenterology and Hepatology, Ospedali Riuniti University Hospital, Ancona, Italy; ‖‖‖‖ Division of Hepatology and Liver Transplantation, California Pacific Medical Center, San Francisco, California; ¶¶¶¶ Department of Surgery, Oncology and Gastroenterology, University of Padua, Padua, Italy; #### Epatocentro Ticino, Lugano, Switzerland; ∗∗∗∗∗ Department of Statistics, University of Oxford, Oxford, United Kingdom

**Keywords:** Cholestatic Liver Disease, Ulcerative Colitis, Crohn’s Disease, Integrin alpha4beta7, ALP, alkaline phosphatase, ALT, alanine transaminase, AST, aspartate aminotransferase, CD, Crohn’s disease, IBD, inflammatory bowel disease, IQR, interquartile range, LT, liver transplantation, PSC, primary sclerosing cholangitis, UC, ulcerative colitis, UDCA, ursodeoxycholic acid, ULN, upper limit of normal

## Abstract

**Background & Aims:**

Gut-homing lymphocytes that express the integrin α4β7 and CCR9 might contribute to development of primary sclerosing cholangitis (PSC). Vedolizumab, which blocks the integrin α4β7, is used to treat patients with inflammatory bowel diseases (IBD), but there are few data on its efficacy in patients with PSC. We investigated the effects of vedolizumab in a large international cohort of patients with PSC and IBD.

**Methods:**

We collected data from European and North American centers participating in the International PSC Study Group from patients with PSC and IBD who received at least 3 doses of vedolizumab (n = 102; median vedolizumab treatment duration, 412 days). Demographic and clinical data were collected from baseline and during the follow-up period (until liver transplantation, death, or 56 days after the final vedolizumab infusion). We analyzed overall changes in biochemical features of liver and proportions of patients with reductions in serum levels of alkaline phosphatase (ALP) of 20% or more, from baseline through last follow-up evaluation. Other endpoints included response of IBD to treatment (improved, unchanged, or worsened, judged by the treating clinician, as well as endoscopic score) and liver-related outcomes.

**Results:**

In the entire cohort, the median serum level of ALP increased from 1.54-fold the upper limit of normal at baseline to 1.64-fold the upper limit of normal at the last follow-up examination (*P* = .018); serum levels of transaminases and bilirubin also increased by a small amount between baseline and the last follow-up examination. Serum levels of ALP decreased by 20% or more in 21 patients (20.6%); only the presence of cirrhosis (odds ratio, 4.48; *P* = .019) was independently associated with this outcome. Of patients with available endoscopic data, 56.8% had a response of IBD to treatment. Liver-related events occurred in 21 patients (20.6%), including bacterial cholangitis, cirrhosis decompensation, or transplantation.

**Conclusions:**

In an analysis of patients with PSC and IBD in an international study group, we found no evidence for a biochemical response to vedolizumab, although serum level of ALP decreased by 20% or more in a subset of patients. Vedolizumab appears to be well tolerated and the overall response of IBD was the same as expected for patients without PSC.

See editorial on page 51.

**What You Need to Know**

**Background**

Aberrant expression of adhesion molecules in the liver and abnormal lymphocyte trafficking are thought to be involved in the pathogenesis of primary sclerosing cholangitis (PSC). Few studies have evaluated the effects of integrin inhibitors such as vedolizumab in patients with PSC.

**Findings**

In analyses of serum samples from patients in a large international uncontrolled study, we found that patients with PSC and IBD treated with vedolizumab had a small increase in liver enzymes and bilirubin levels at end of the follow-up period. However, one-fifth had a significant reduction in level of alkaline phosphatase regardless of use of ursodeoxycholic acid. The endoscopic response of IBD in patients with PSC and IBD did not differ significantly from that reported for patients with only IBD.

**Implications for patient care**

Although we did not find evidence for a biochemical response to vedolizumab in patients with IBD and PSC, the response was heterogeneous—a subset of patients might benefit from therapy. Vedolizumab seems to be safe in patients with PSC and IBD.

The close association of primary sclerosing cholangitis (PSC) with inflammatory bowel disease (IBD) has long suggested that common pathophysiological mechanisms acting in the liver and intestine could be found and targeted therapeutically. Although liver disease and intestinal inflammation in PSC can progress along apparently independent courses, they also influence one another, such as increased post-transplant PSC recurrence in patients who have a pouch or intact colon^1^ and the increased risk of colorectal cancer in patients with IBD with PSC as compared with IBD alone.^2^ Furthermore, the phenotype of IBD in PSC has particular characteristics, such as involvement of the entire colon with right-sided dominance, ileal inflammation, and relative rectal sparing. Some investigators suggest that the IBD associated with PSC is its own distinct entity separate from ulcerative colitis (UC) or Crohn’s disease alone (CD).^3^, ^4^, ^5^, ^6^ However, despite recent advances in the characterization of PSC and the IBD associated with PSC, no proven beneficial medical therapy is available to slow the progression to advanced liver disease and/or malignancy, and the prognosis of patients with PSC remains guarded.^7^

In contrast, there are several effective treatments for IBD, including recently developed targeted biologic therapies. One such is vedolizumab, which blocks the α4β7 integrin, and is effective in CD and UC.^8^ Several studies suggest that the particular gut-homing pathway that vedolizumab targets (namely the interaction between α4β7 and its ligand, mucosal addressin cellular adhesion molecule-1) is implicated in the pathophysiology of PSC.^9^, ^10^, ^11^ This includes overexpression of mucosal addressin cellular adhesion molecule-1 in the PSC hepatic endothelial cells, and related integrins and chemokine ligands, which can promote α4β7/mucosal addressin cellular adhesion molecule-1 interactions, such as vascular adhesion protein-1 and CC-chemokine ligand 25, respectively.^10^, ^12^ Therefore, it is possible that vedolizumab may play a role in reducing lymphocyte infiltration into the liver in patients with PSC and thereby in reducing hepatic and biliary inflammation. Indeed, it has been shown that vedolizumab can induce clinical remission in rheumatologic extraintestinal manifestations of IBD.^13^

This hypothesis has not been tested in clinical trials, although a few observational studies published recently document the clinical experience of treating patients who have PSC and IBD with vedolizumab.^14^ These cohorts are usually limited to a small number of centers, leading to a small sample size. We sought to contribute to this literature by documenting our experience in a larger international cohort of patients with PSC and IBD.

## Methods

### Patient Cohort

A retrospective analysis was carried out on patients with PSC and IBD receiving vedolizumab as indicated for their IBD (UC, CD, or IBD-unspecified). Investigators from 20 centers across Europe and North America who are active members of the International PSC Study Group contributed patient data (Supplementary Table 1). To be eligible for inclusion in the dataset, patients must have been diagnosed with PSC according to internationally accepted guidelines,^15^ have received a minimum of 3 doses of vedolizumab for their IBD, have baseline (prevedolizumab) and follow-up blood tests including liver biochemistry, have commenced vedolizumab with their native liver still *in situ*, and received vedolizumab according to the usual dosing schedule as licensed.^16^

### Outcomes

The effect of vedolizumab on progression of PSC was evaluated by analyzing the change in liver biochemistry from baseline to various time points when on vedolizumab. This was done in 2 ways.

First, we analyzed the overall change in alkaline phosphatase (ALP), alanine aminotransferase (ALT), aspartate aminotransferase (AST), and bilirubin levels at baseline, Week 6 (ie, Day 42), Week 14 (ie, Day 96), and last follow-up while on vedolizumab. For further details on the time-points collected, see the Supplementary Methods.

Second, we deduced the proportion of patients whose ALP dropped by 20% or more from baseline to last follow-up. This proportionate drop was chosen because it was believed to represent a drop of ALP of a larger magnitude than what would usually be considered to occur as part of the natural history of the disease and therefore considered clinically significant.^17^ We also calculated the proportion of patients whose ALP rose by 20% from baseline to last follow-up, and those whose ALP remained stable (ie, ± 20%).

We sought to collect endoscopic IBD response, classified as improved, or unchanged/worsened (as judged by the treating clinician), and endoscopic scores at baseline and on vedolizumab where available, namely the Mayo Endoscopic Subscore,^18^ the Ulcerative Colitis Endoscopic Index of Severity,^19^ and the Simple Endoscopic Score for Crohn’s Disease.^20^ We also looked at whether there was an association between endoscopic IBD response and change in ALP at last follow-up using the univariate analyses described later.

Finally, we collected liver-related outcomes, which included any of the following: listing for liver transplantation (LT), undergoing LT, ascending cholangitis, new-onset ascites, variceal bleed, hepatic encephalopathy, cholangiocarcinoma, and death.

### Statistical Analyses

Paired Student *t* tests or Wilcoxon matched-pairs signed rank tests were used according to whether the data was distributed parametrically or nonparametrically, respectively. Univariate logistic regression and multivariate logistic regression were carried out to assess the impact of relevant variables on ALP changes from baseline to last follow-up. The Supplementary Methods provide detailed information on statistical analyses.

## Results

### Baseline Demographics

Of 133 patients whose data were contributed, 102 patients met inclusion criteria for the study. Reasons for exclusion were incomplete ALP data (n = 15), first dose of vedolizumab received after LT (n = 13), and less than 3 doses of vedolizumab administered (n = 3). Table 1 summarizes baseline demographics, clinical, and laboratory information for the 102 study subjects: 64/102 (62.8%) were male, and most patients had classical large-duct PSC (90.2%). One-fifth of patients had cirrhosis at baseline, and most patients had associated UC (64.7%).

**Table 1:** Baseline Demographics, Clinical, and Laboratory Data (n = 102)

Male, n (*%*)	64 (62.8)
Age at PSC diagnosis, mean ± SD (range), *y*	31.4 ± 14.2 (11–86)
Age at IBD diagnosis, mean ± SD (range), *y*	26.0 ± 12.3 (9–62)
Cirrhosis, n (*%*)	21 (20.6)
Type of PSC, n (*%*)	
Large-duct PSC	92 (90.2)
Small-duct PSC	8 (7.8)
PSC/AIH overlap	2 (2.0)
Type of IBD, n (*%*)	
Ulcerative colitis	66 (64.7)
Crohn’s disease	30 (29.4)
IBD-unspecified	6 (5.9)
UDCA use, n (*%*)	61 (59.8)
Mean dose ± SD (range), *mg/kg/day*	13.9 ± 3.7 (5–20.4)
Previous anti-TNF use, n (*%*)	66 (64.7)
Duration of vedolizumab, median (range), *d*	412 (37–2609)
ALP >ULN at baseline, n (*%*)	69 (67.7)
Baseline laboratory tests, median (IQR)	
Alkaline phosphatase (*IU/L × ULN*)	1.54 (0.86–2.67)
Alanine transaminase (*IU/L*)	38 (22–76)
Aspartate transaminase (*IU/L*)	38 (23–69)
Bilirubin (*μmol/L*)	10 (6.6–16.0)
Albumin (*g/L*)	37 (33–41)
Platelets (*x10*^*6*^*/L*)	330 (213–401)
International normalized ratio	1 (0.97–1.12)
Creatinine (*μmol/L*)	70 (63–80)
Sodium (*μmol/L*)	139 (137–141)

AIH, autoimmune hepatitis; ALP, alkaline phosphatase; IBD, inflammatory bowel disease; IQR, interquartile range; PSC, primary sclerosing cholangitis; SD, standard deviation; TNF, tumor necrosis alpha; UDCA, ursodeoxycholic acid; ULN, upper limit of normal.

The median duration of vedolizumab treatment was 412 days (interquartile range [IQR], 180–651; range, 37–2609). See Supplementary Results for further information re vedolizumab duration, follow-up, and time-points of last follow-up liver biochemistry variables. Forty-four patients discontinued use of vedolizumab, predominantly for lack of efficacy (72.3%) and adverse events (13.6%).

### Changes in Liver Chemistry

**Figure 1:**
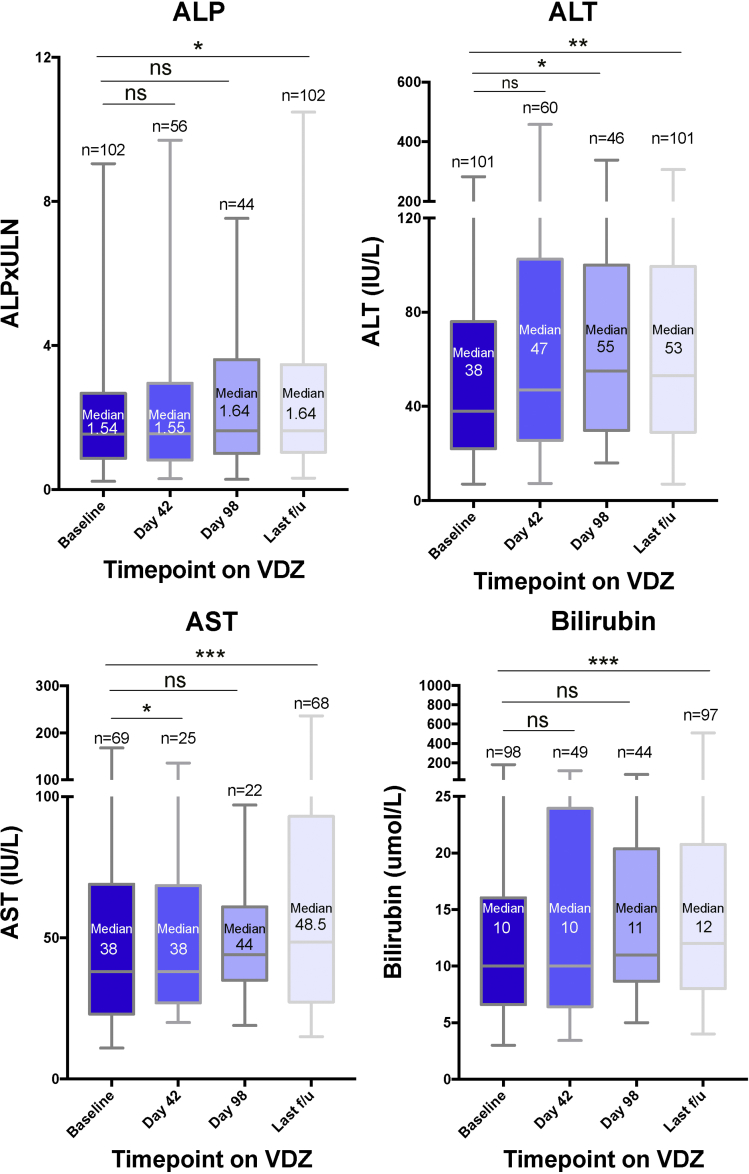
Change in liver biochemistry over time on vedolizumab. The median, IQR, and range are shown. The number of patients with liver biochemistry values and therefore included in the analyses are shown for each timepoint above the corresponding box and whiskers plot. Wilcoxon matched-pairs signed rank test was performed. VDZ, vedolizumab. ns = *P* > .05; *P ≤ .05; **P ≤ .01; ***P ≤ .001.

In the total cohort, there was a small increase in liver enzymes over time (Figure 1). The median ALP gradually increased from baseline (1.54 × upper limit of normal [ULN]; IQR, 0.86–2.67) to last follow-up (1.64 × ULN; IQR, 1.04–3.47; *P* = .018), with similar to intermediate values at Week 6 (1.55 × ULN; IQR, 0.82–2.95; *P* = .084) and Week 14 (1.64 × ULN; IQR, 1.00–3.61; *P* = .52). A comparable increase was seen for median ALT (baseline, 38 IU/L; IQR, 22–76 vs last follow-up, 53 IU/L; IQR, 29–100; *P* = .002), median AST (baseline, 38 IU/L; IQR, 23–69 vs last follow-up, 49 IU/L; IQR, 27–93; *P* = .0002), and median bilirubin (baseline, 10 μmol/L; IQR, 7–16 vs last follow-up, 12 μmol/L; IQR, 8–21; *P* = .0002).

**Figure 2:**
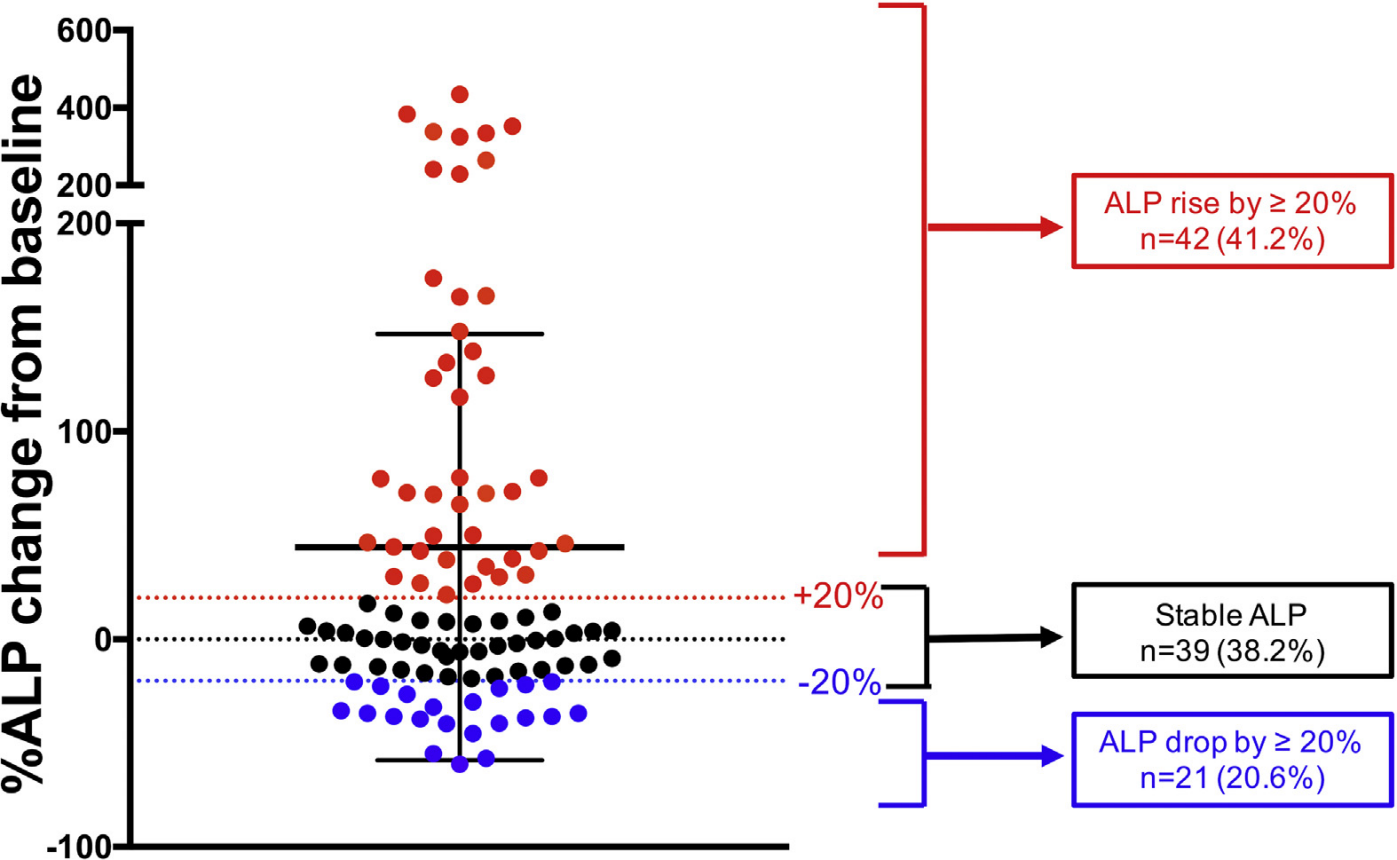
The percentage change in ALP from baseline to last follow-up. Each *dot* represents an individual patient (n = 102) and is color coded to show 3 different groups. *Red*, ALP increase by ≥20%; *black*, stable ALP (-20% to +20%); *blue*, ALP drop by ≥20%. The *black dotted line* at 0 represents no change, with those below having a decrease in ALP at last follow-up and those above having an increase in ALP at last follow-up, as compared with baseline ALP before vedolizumab.

**Supplementary Figure 1:**
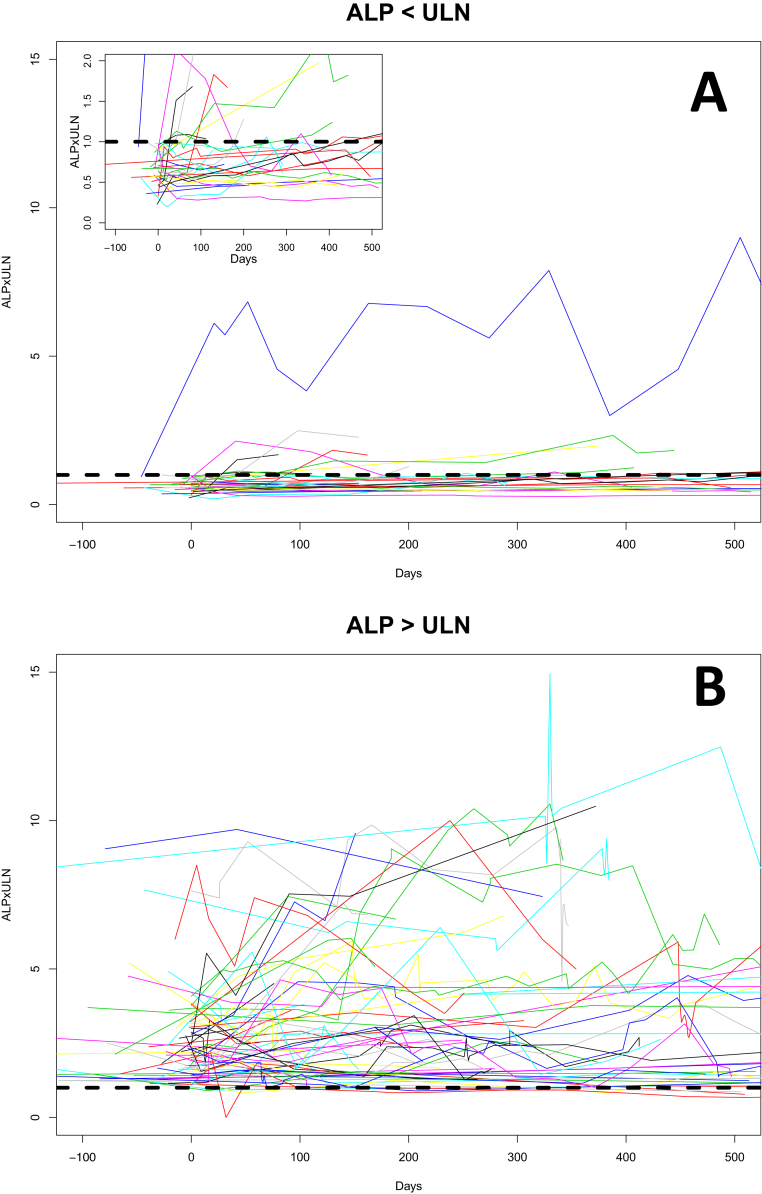
The trajectory of ALP from baseline over time according to baseline ALP below and above ULN (n = 102). These spaghetti plots show ALP at baseline and different time points on vedolizumab. Each *line* represents a unique patient. The *dotted black line* represents an ALP of 1 × ULN. (*A*) Patients whose baseline ALP was less than 1 × ULN. (*B*) Patients whose baseline ALP was greater than 1 × ULN. The *inset* in *A* is a magnified portion of the graph to more easily see the trajectory of those below the ULN. Vedolizumab was started at Day 0. Patients whose baseline ALP was <ULN tended to follow a fairly stable course, whereas those with baseline >ULN followed a fairly erratic course.

Twenty-one (20.6%) patients had an ALP drop ≥20% from baseline to last follow-up. Thirty-nine patients (38.2%) had a stable ALP, whereas 42 patients (41.2%) had ALP increase by ≥20% at last follow-up (Figure 2). The trajectories of the ALP from baseline over time are shown in Supplementary Figure 1.

On univariate analysis, the presence of cirrhosis was associated with an ALP drop of ≥20% from baseline to last follow-up (odds ratio, 4.70; 95% confidence interval, 1.61–13.76) (Table 2). This finding was reproduced on multivariate analysis. No other variables were associated with ≥20% ALP drop, including ursodeoxycholic acid (UDCA) use at baseline. However, we observed a trend toward an association with a raised baseline ALP, and having CD or IBD-unspecified rather than UC. Twenty-nine percent of female patients and 42.9% of patients with cirrhosis achieved such drop in ALP compared with 15.6% of males and 13.8% of patients without cirrhosis. Of note, only 3 of the 21 patients with an ALP drop ≥20% had a normal ALP at baseline. No variables were associated with ALP increase ≥20% from baseline (Supplementary Table 3).

**Table 2:** Univariate and Multivariate Analysis for ALP Drop by 20% or More From Baseline to Last Follow-up

Variable	Univariate		Multivariate	
	Odds ratio (95% CI)	*P* value	Odds ratio (95% CI)	*P* value
Cirrhosis	4.70 (1.61–13.76)	.005	4.48 (1.28–15.72)	.019
Baseline ALP >ULN^a^	3.53 (0.96–12.98)	.058	3.39 (0.76–15.25)	.111
Ulcerative colitis^b^	0.37 (0.12–1.19)	.096	0.35 (0.10–1.19)	.092
Male gender	0.46 (0.17–1.20)	.112	0.55 0.17–1.72)	.301
Age at diagnosis of PSC^c^	1.03 (0.99–1.06)	.111	1.01 (0.97–1.05)	.723
UDCA use at baseline	0.68 (0.26–1.79)	.438	0.55 (0.17–1.78)	.318
Small-duct PSC^d^	0.55 (0.06–4.74)	.585	—	
PSC-AIH overlap^d^	3.84 (0.23–64.29)	.349	—	
Duration vedolizumab^e^	1.04 (0.98–1.09)	.178	—	
IBD improvement on vedolizumab^f^	1.18 (0.37–3.75)	.777	—	
Previous anti-TNF use	0.63 (0.22–1.83)	.395	—	

AIH, autoimmune hepatitis; ALP, alkaline phosphatase; CI, confidence interval; IBD, inflammatory bowel disease; PSC, primary sclerosing cholangitis; TNF, tumor necrosis factor; UDCA, ursodeoxycholic acid; ULN, upper limit of normal.

a Baseline indicating last ALP taken before vedolizumab commenced.

b Versus IBD-unspecified or Crohn’s disease.

c Per 1-year increase.

d Versus large-duct PSC.

e Per 1-month increase in vedolizumab duration.

f Endoscopic improvement versus unchanged/worsened.

Refer to Supplementary Results for additional analyses carried out on specific cohorts, including patients with cirrhosis, those not on UDCA, those who had been on vedolizumab for a minimum of 6 months, and those with baseline ALP >1.5 × ULN.

### Endoscopic Inflammatory Bowel Disease Response

Data on endoscopic response to vedolizumab at baseline and at follow-up were available for 74 patients. The median duration from vedolizumab initiation to follow-up endoscopy was 227 days (IQR, 130–336; range, 67–2540). Forty-two patients (56.8%) had an endoscopic IBD response, with the remainder worsened or unchanged. Normal ALP at baseline and longer vedolizumab duration were associated with an endoscopic IBD response, although type of IBD was not associated (Supplementary Table 6).

**Figure 3:**
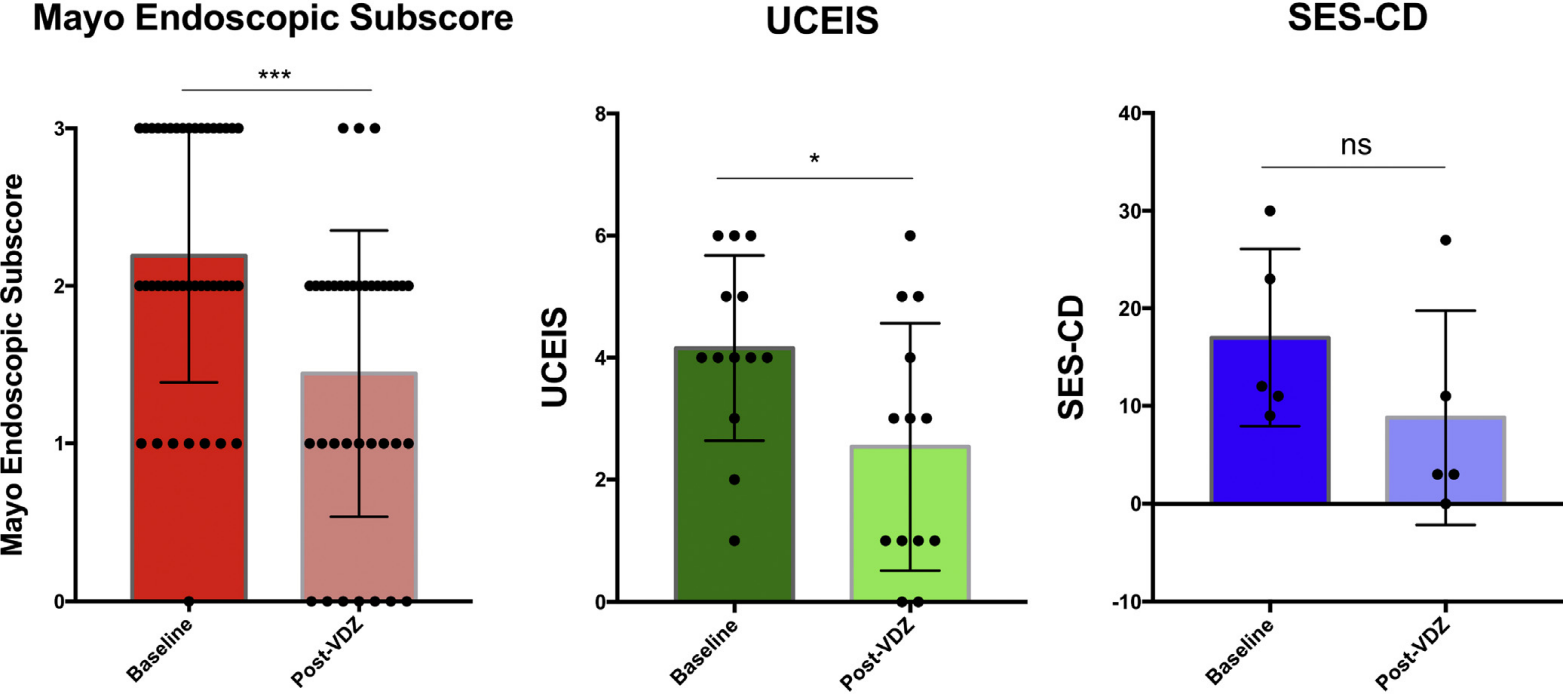
Endoscopic IBD response at baseline endoscopy before vedolizumab and after treatment with vedolizumab. Paired endoscopic scores were available for 36 patients for Mayo Endoscopic Subscore, 13 patients for UCEIS, and 5 patients for SES-CD. Individual scores for each patient are shown as *black dots*, with *bars* indicating the mean value and the standard deviation is shown. Paired Student *t* test performed. SES-CD, Simple Endoscopic Score for Crohn’s Disease; UCEIS, Ulcerative Colitis Endoscopic Index of Severity; VDZ, vedolizumab; ns = *P* > .05; *P ≤ .05; ***P ≤ .001.

Endoscopic scores were available for a subset of patients (Figure 3). Among patients with UC, the mean endoscopic scores dropped prevedolizumab versus postvedolizumab for Mayo Endoscopic Subscore (2.2 ± 0.8 to 1.4 ± 0.9; *P* = .0008) and Ulcerative Colitis Endoscopic Index of Severity (4.2 ± 1.5 to 2.5 ± 2.0; *P* = .028). Among patients with CD, Simple Endoscopic Score for Crohn’s Disease prevedolizumab and postvedolizumab was only available for 5 patients and there was a numerical improvement in mean Simple Endoscopic Score for Crohn’s Disease (17.0 ± 9.1 to 8.8 ± 11.0; *P* = .215).

### Safety and Liver-Related Outcomes

Safety and liver-related outcomes were calculated for the 102 patients described previously, and the 3 patients who had received fewer than 3 vedolizumab infusions and had follow-up liver biochemistry data (1 infusion [n = 2]) and 2 infusions [n = 1]). Of these 105 patients, a 3-fold elevation in ALP, ALT, and AST from baseline to last follow-up was observed in 6 (5.7%), 11 (10.4%), and 3 (2.9%) patients; doubling of total bilirubin was noted in 21 (20.0%).

Twenty-two patients (20.9%) experienced a liver-related outcome over the median follow-up period of 561 days. Twelve patients (11.4%) were listed for LT, of whom 8 (7.6%) underwent LT. Nine patients (8.8%) experienced at least 1 episode of cholangitis and 6 patients (5.9%) had new-onset ascites. No patient experienced a variceal bleed, nor developed cholangiocarcinoma, and there were no deaths. On univariate analysis, cirrhosis, baseline ALP >ULN, and baseline albumin level were associated with the occurrence of a liver-related outcome (Supplementary Table 7). Among patients with cirrhosis (n = 21), 3/9 (33.3%) of patients who had an ALP drop ≥20% had a liver-related complication, compared with 7/12 (58.3%) who did not have an ALP drop ≥20%.

## Discussion

The data presented here, which represent an international, multicenter experience, add substantially to the existing literature on the subject of patients with PSC exposed to vedolizumab. The demographics of the cohort closely resembled that reported in the literature, with most having large-duct PSC, two-thirds having UC, and one-third CD or IBD-unspecified, a proportion having advanced liver disease, and just under two-thirds being on UDCA treatment.^17^, ^21^, ^22^ The median age at PSC diagnosis of 31.4 years is slightly lower than in the literature (40 years),^23^ which may reflect the demographic who have more active IBD or are more likely to receive a biologic therapy. However, one should keep in mind that this cohort has active IBD, which in itself is a minority of the PSC demographic, because most patients with PSC have fairly quiescent IBD, not requiring biologic therapy (or, 20%–40% do not have IBD at all).^5^ Therefore, the effect of vedolizumab in PSC with inactive IBD or no IBD has never been observed or evaluated.

It is immediately apparent that there is heterogeneity with regard to changes in ALP, an important marker of cholestasis. The reduction in ALP of ≥20% from baseline in a subset of treated patients may indicate a biologic effect of vedolizumab in patients with PSC, although a more pronounced spontaneous fluctuation cannot be excluded. ALP is thought to be a potential surrogate marker for clinical outcome in PSC, and has been proposed by a panel of experts as an important endpoint in clinical trials.^24^ Although spontaneous normalization of ALP has been described in a variable proportion of patients with PSC irrespective of endoscopic intervention or use of UDCA,^25^ the natural history of PSC is for the ALP level to remain relatively stable or increase slightly over time. For instance, in a recent clinical trial investigating the role of norUDCA on patients with PSC, the 40 patients on placebo had a mean relative change of 1.2% increase over the 12-week study duration.^17^ Therefore, the observed reduction in ALP by ≥20% in a subset of patients on vedolizumab suggests a possible therapeutic signal worthy of additional investigation. However, a drop in ALP does not necessarily correlate with improved clinical outcome. As seen in the high-dose UDCA trial for patients with PSC, those on UDCA experienced a significant drop in ALP as compared with placebo, but developed more liver-related clinical endpoints.^26^

In the cohort overall, the median values for ALP, AST, ALT, and bilirubin all increased from baseline to last follow-up on vedolizumab. This increase, however, was small, and may reflect changes commensurate with the natural history and progression of the disease. Median ALP rose from 1.54 × ULN to 1.64 × ULN, which is equivalent to 184 to 197 IU/L (if ULN = 120 IU/L). This is itself a clinically insignificant increase, and therefore, when viewed overall, the increase in ALP is unlikely to represent a significant safety signal.

The drop in ALP of ≥20% was associated with cirrhosis and there was a trend toward an association with a higher ALP at baseline. Patients with cirrhosis and those with a raised ALP are potentially more likely to have a more inflammatory and progressive form of PSC, and therefore may theoretically benefit more substantially from an anti-inflammatory effect of reduced migration of lymphocytes to the liver. Furthermore, vedolizumab is degraded to smaller peptides and amino acids to be excreted by the kidneys, and this process is partially done by hepatic proteolytic degradation.^27^ Cirrhosis may reduce/slow the breakdown of vedolizumab thereby leading to higher serum concentrations, possibly leading to greater clinical effect.

It is difficult to conclude whether this association of cirrhosis with ALP reduction ≥20% is a true finding or a spurious one. Within that cohort of patients with cirrhosis, we could not show that changes in ALP correlated with changes in liver synthetic function, such as bilirubin and international normalized ratio. In any case, there was no clear indication that patients with cirrhosis did worse on vedolizumab, and should a future prospective trial be carried out in PSC, it would be important to include patients with compensated cirrhosis and explore this association further.

Christensen et al^28^ also observed an association of an elevated ALP at baseline with subsequent reduction after treatment with vedolizumab. In their study, when examining patients with raised ALP at baseline, 11/18 patients (69%) had an ALP drop, with median ALP going from 475 IU/L at baseline to 322.5 IU/L at Week 14 and 283 IU/L at Week 30. In patients with normal ALP at baseline, there was a trend for the ALP to increase slightly. Equally, these findings should be interpreted with caution, because statistical regression to the mean may account for the association with a higher ALP at baseline, and we were unable in this retrospective study to distinguish hepatic, intestinal, and other isoforms of ALP.

One fifth of our cohort (22/105) experienced a liver-related outcome. This may be slightly overrepresented by the occurrence of cholangitis (8.8%), which in itself, unless recurrent and refractory to oral antibiotics, is not necessarily an indication of advanced liver disease requiring LT. If we exclude those with cholangitis only (7/9), then 14/102 (13.7%) experienced a liver-related outcome. These findings are similar to the recently reported trial of anti-LOXL-2 antibody, simtuzumab, in PSC, where 47/234 patients (20.1%) experienced a PSC-related event, with an overrepresentation of cholangitis (n = 31, or 13.2% of total cohort).^29^ This proportion of liver-related outcomes is consistent with the natural history of PSC and does not by itself indicate that vedolizumab treatment is harmful in PSC.

It is important to highlight the fact that a significant proportion of patients had an endoscopic IBD response, both as reported by the treating clinician (42/74 patients; 56.8%) and by the more robust measure of drop in individual endoscopic scores (particularly Mayo Endoscopic Subscore and Ulcerative Colitis Endoscopic Index of Severity). Such a response rate is similar to that found in IBD alone.^8^ Given the possibility that PSC-IBD represents an immunologically and phenotypically distinct form of IBD from IBD alone,^3^, ^4^, ^5^, ^6^ this finding is reassuring for clinicians considering vedolizumab to treat active IBD in patients with PSC.

A major limitation of this study is its retrospective nature. The use of objective outcome measures, such as liver biochemistries and endoscopic scores, serves to minimize bias; however, adverse events, particular those not leading to hospitalization, death, or LT could potentially be underreported. In addition, the absence of a comparator group, such as a placebo-treated, or matched control cohort, is a weakness that disallows attribution of causality. Finally, we did not collect information on UDCA use after baseline. Therefore, some patients might have started UDCA after starting vedolizumab, which could conceivably alter the ALP values at follow-up. Nevertheless, we separately examined the subgroup of patients not on UDCA at baseline, and observed similar changes in ALP and other liver biochemistries compared with patients taking UDCA, suggesting that we cannot attribute the observed changes to the use of UDCA.

In conclusion, this large international experience of vedolizumab in patients with PSC and IBD shows that a clear-cut biochemical response to vedolizumab is not observed across the entire cohort. However, a subset of patients had a substantial drop in their ALP of 20% or more. Patients with more aggressive disease, such as the presence of cirrhosis and potentially those with a raised ALP at baseline, were more likely to respond. In addition, the proportion of patients experiencing a liver-related outcome seems to be in keeping with the natural history of disease. Furthermore, more than half of patients with IBD and PSC treated with vedolizumab had an endoscopic IBD response, similar to rates reported in IBD-only patients, and without an apparent increase in the discontinuation rate. Despite the disappointment with lack of a uniform response, further evaluation of vedolizumab as a beneficial treatment in PSC may be warranted in a subset of patients via a stratified randomized clinical trial.

## Figures and Tables

**Supplementary Table 1:** Participating Center and the Number of Patients From Each Center Included in the Overall Cohort (n = 102)

Center	Location	n
Oxford University Hospitals	Oxford, UK	15
Friedrich-Alexander-University	Erlangen, Germany	10
University of Alberta	Edmonton, Canada	10
Academic Medical Center	Amsterdam, Netherlands	7
University Hospitals Birmingham	Birmingham, UK	7
University Medical Center Hamburg-Eppendorf	Hamburg, Germany	7
University of Miami	Miami, FL	6
Massachusetts General Hospital	Boston, MA	6
University of California Davis	Davis, CA	6
Karolinska Instituet	Stockholm, Sweden	6
University of Washington Medical Center	Seattle, WA	4
Yale University School of Medicine	New Haven, CT	4
Erasmus Medical Center	Rotterdam, Netherlands	4
University of Copenhagen	Copenhagen, Denmark	3
Medical University of Vienna	Vienna, Austria	2
California Pacific Medical Center	San Francisco, CA	1
University of Gothenburg	Gothenburg, Sweden	1
Ospedali Riuniti University Hospital	Ancona, Italy	1
University of Padova	Padova, Italy	1
Epatocentro Ticino	Lugano, Switzerland	1

**Supplementary Table 2:** Number of Variables Missing Within the Complete Dataset (n = 102)

Variable	N
Presence of cirrhosis	1
Baseline ALP	0
Type of IBD	0
Gender	0
Age at diagnosis of PSC	0
UDCA use at baseline	0
Type of PSC	0
Duration vedolizumab	0
IBD response	28
Previous anti-TNF use	7
Paired baseline follow-up ALP	0
Paired baseline and follow-up ALT	1
Paired baseline and follow-up AST	34
Paired baseline and follow-up bilirubin	5
Baseline platelets	3
Baseline INR	39
Baseline albumin	9

ALP, alkaline phosphatase; ALT, alanine aminotransferase; AST, aspartate aminotransferase; IBD, inflammatory bowel disease; INR, international normalized ratio; PSC, primary sclerosing cholangitis; TNF, tumor necrosis factor; UDCA, ursodeoxycholic acid.

**Supplementary Table 3:** Univariate and Multivariate Analysis for ALP Rise by 20% or More From Baseline to Last Follow-up

Variable	Univariate		Multivariate	
	OR (95% CI)	*P* value	OR (95% CI)	*P* value
Cirrhosis	0.20 (0.02–1.60)	.130	0.163 (0.02–1.44)	.103
Baseline ALP >ULN^a^	0.47 (0.16–1.35)	.161	0.78 (0.23–2.62)	.679
Ulcerative colitis^b^	2.96 (0.79–11.10)	.107	2.66 (0.67–10.54)	.165
Male gender	1.52 (0.49–4.72)	.466	1.52 (0.45–5.19)	.502
Age at diagnosis of PSC^c^	1.01 (0.97–1.04)	.767	1.02 (0.98–1.06)	.416
UDCA use at baseline	0.54 (0.19–1.53)	.244	0.56 (0.17–1.91)	.358
Small-duct PSC^d^	1.71 (0.31–9.30)	.534	—	
PSC-AIH overlap^d^	n/a^e^	n/a	—	
Duration vedolizumab^f^	1.01 (0.95–1.06)	.832	—	
IBD improved^g^	0.90 (0.24–3.26)	.873	—	
Previous anti-TNF use	1.53 (0.45–5.18)	.492	—	

AIH, autoimmune hepatitis; ALP, alkaline phosphatase; CI, confidence interval; IBD, inflammatory bowel disease; n/a, not applicable; OR, odds ratio; PSC, primary sclerosing cholangitis; TNF, tumor necrosis factor; UDCA, ursodeoxycholic acid; ULN, upper limit of normal.

a Baseline indicating last ALP taken before vedolizumab commenced.

b Versus IBD-unspecified or Crohn’s disease.

c Per 1-year increase.

d Versus large-duct PSC.

e There were too few numbers of events to fit the binomial generalized linear model, and therefore an OR could not be calculated.

f Per 1-month increase in vedolizumab duration.

g Endoscopic improvement versus unchanged/worsened.

**Supplementary Table 4:** Univariate and Multivariate Analysis for ALP Drop by 20% or More From Baseline to Last Follow-up Among Patients Not on Ursodeoxycholic Acid (n = 41)

Variable	Univariate		Multivariate	
	Odds ratio (95% CI)	*P* value	Odds ratio	*P* value
Cirrhosis	7.80 (1.60–38.11)	.011	5.72 (0.68–47.79)	.107
Baseline ALP >ULN^a^	2.83 (0.62–13.04)	.181	2.12 (0.27–16.34)	.472
Ulcerative colitis^b^	0.24 (0.05–1.10)	.067	0.41 (0.06–2.92)	.370
Male gender	0.42 (0.10–1.81)	.245	0.42 (0.06–3.03)	.392
Age at diagnosis of PSC^c^	1.06 (1.00–1.12)	.053	1.03 (0.95–1.11)	.468
Small-duct PSC^d^	1.75 (0.14–21.88)	.664	—	
PSC-AIH overlap^d^	3.50 (0.20–62.42)	.394	—	
Duration vedolizumab^e^	1.06 (0.98–1.14)	.157	—	
IBD improved^f^	0.75 (0.14–4.13)	.741	—	
Previous anti-TNF use	0.83 (0.19–3.75)	.812	—	

AIH, autoimmune hepatitis; ALP, alkaline phosphatase; CI, confidence interval; IBD, inflammatory bowel disease; PSC, primary sclerosing cholangitis; TNF, tumor necrosis factor; ULN, upper limit of normal.

a Baseline indicating last ALP taken before vedolizumab commenced.

b Versus IBD-unspecified or Crohn’s disease.

c Per 1-year increase.

d Versus large-duct PSC.

e Per 1-month increase in vedolizumab duration.

f Endoscopic improvement versus unchanged/worsened.

**Supplementary Table 5:** Univariate Analysis for ALP <1.5 × ULN at Last Follow-up, Among Subgroup of Patients for Whom Baseline ALP >1.5 × ULN (n = 51)^a^

Variable	Univariate	
	OR (95% CI)	*P* value
Cirrhosis	0.62 (0.11–3.38)	.576
Ulcerative colitis^b^	0.07 (0.01–0.39)	.002
Male gender	0.34 0.08–1.43)	.142
Age at diagnosis of PSC^c^	1.02 (0.97–1.08)	.434
UDCA use at baseline	2.56 (0.48–13.62)	.270
Small-duct PSC^d^	n/a^e^	n/a
PSC-AIH overlap^d^	n/a^e^	n/a
Duration vedolizumab^f^	1.04 (0.95–1.13)	.411
IBD improved^g^	0.92 (0.17–4.86)	.925
Previous anti-TNF use	0.30 (0.07–1.33)	.112

AIH, autoimmune hepatitis; ALP, alkaline phosphatase; CI, confidence interval; IBD, inflammatory bowel disease; n/a, not applicable; OR, odds ratio; PSC, primary sclerosing cholangitis; TNF, tumor necrosis factor; UDCA, ursodeoxycholic acid; ULN, upper limit of normal.

a Baseline indicating last ALP taken before vedolizumab commenced.

b Versus IBD-unspecified or Crohn’s disease.

c Per 1-year increase.

d Versus large-duct PSC.

e There were too few numbers of events to fit the binomial generalized linear model, and therefore an OR could not be calculated.

f Per 1-month increase in vedolizumab duration.

g Endoscopic improvement versus unchanged/worsened.

**Supplementary Table 6:** Univariate Analysis for Endoscopic IBD Response (n = 74)

Variable	Univariate	
	OR (95% CI)	*P* value
Cirrhosis	0.79 (0.27–2.35)	.677
Baseline ALP >ULN^a^	0.29 (0.10–0.85)	.024
Ulcerative colitis^b^	1.01 (0.39–2.61)	.979
Male gender	1.12 (0.43–2.90)	.815
Age at diagnosis of PSC^c^	1.01 (0.97–1.05)	.635
UDCA use at baseline	2.57 (0.97–6.81)	.058
Small-duct PSC^d^	1.15 (0.18–7.35)	.880
PSC-AIH overlap^d^	n/a^e^	n/a
Duration vedolizumab^f^	1.08 (1.02–1.14)	.013
Previous anti-TNF use	1.47 (0.53–4.08)	.456

AIH, autoimmune hepatitis; ALP, alkaline phosphatase; CI, confidence interval; IBD, inflammatory bowel disease; n/a, not applicable; OR, odds ratio; PSC, primary sclerosing cholangitis; TNF, tumor necrosis factor; UDCA, ursodeoxycholic acid; ULN, upper limit of normal.

a Baseline indicating last ALP taken before vedolizumab commenced.

b Versus IBD-unspecified or Crohn’s disease.

c Per 1-year increase.

d Versus large-duct PSC.

e There were too few numbers of events to fit the binomial generalized linear model, and therefore an OR could not be calculated.

f Per 1-month increase in vedolizumab duration.

**Supplementary Table 7:** Univariate Analysis for Liver-Related Outcome (n = 102)^a^

Variable	Odds ratio	*P* value
Cirrhosis	5.70 (1.96–16.57)	.001
Baseline ALP >ULN^b^	1.67 (1.23–2.28)	.001
Ulcerative colitis^c^	1.12 (0.40–3.08)	.833
Male gender	1.63 (0.57–4.65)	.358
Age at diagnosis of PSC^d^	0.99 (0.96–1.03)	.696
Baseline serum albumin^b^^,^^e^	0.90 (0.83–0.98)	.024
Baseline platelets^b^^,^^f^	0.96 (0.92–1.00)	.051
Baseline INR^b^^,^^g^	1.28 (0.84–1.95)	.251

ALP, alkaline phosphatase; IBD, inflammatory bowel disease; INR, international normalized ratio; LT, liver transplantation; PSC, primary sclerosing cholangitis; ULN, upper limit of normal.

a Liver-related outcome defined as any of the following: listing for LT, undergoing LT, ascending cholangitis, new-onset ascites, variceal bleed, cholangiocarcinoma, and death.

b Baseline indicating last laboratory value taken before vedolizumab commenced.

c Versus IBD-unspecified or Crohn’s disease.

d Per 1-year increase.

e Per 1 mg/L increase.

f Per 10 × 10^9^/L increase.

g Per 0.1-units increase.

**Supplementary Figure:**
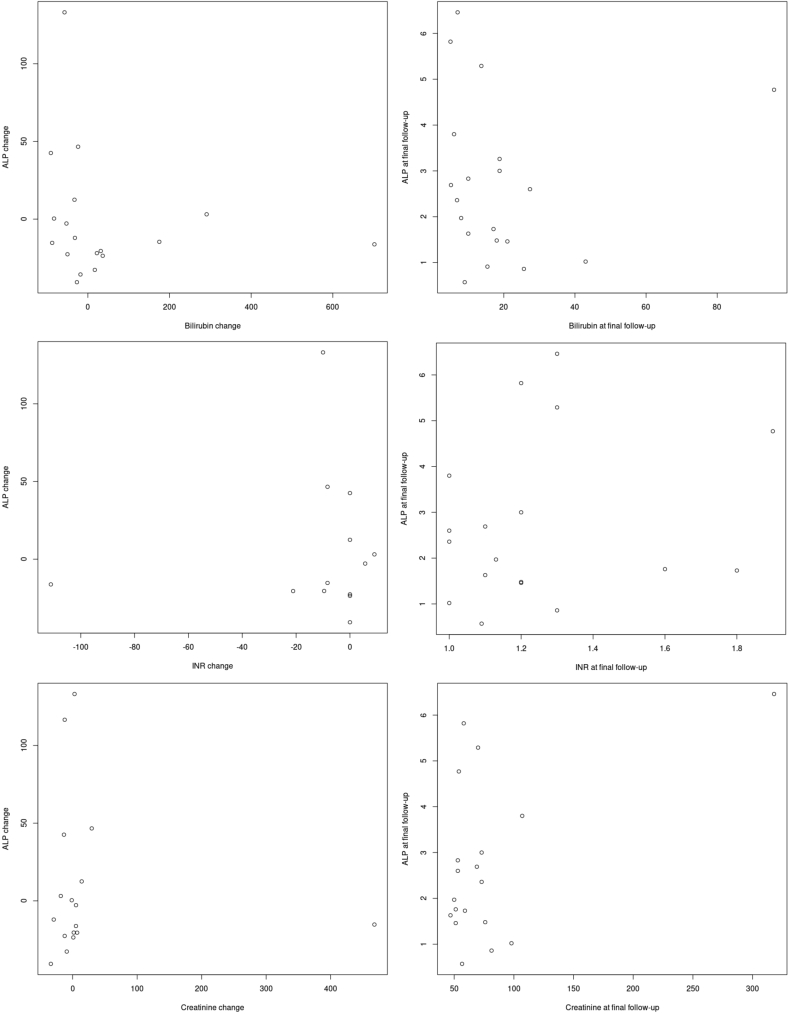


## Supplementary Methods

### Data Collected

A pro forma detailing the inclusion and exclusion criteria, baseline demographics, and necessary data for collection was sent electronically to participating centers, and data were collated centrally (by KDL). Efforts were made to collect missing data by electronic correspondence with participating centers. Patients for whom data were incomplete in respect of treatment or ALP at baseline and follow-up were excluded from the analysis. Endoscopic data were provided where available; however, the lack of endoscopic scoring for IBD before the start of vedolizumab and at last follow-up was not regarded as an exclusion criterion.

Baseline demographics collected included gender, age at diagnosis of PSC, type of PSC, presence of cirrhosis, type of IBD, race, and ethnicity. The presence or not of cirrhosis was determined by the treating clinician according to clinical parameters, imaging, and liver biopsy (where available). Whether vedolizumab had been discontinued was recorded, as was the reason for discontinuation (if known), the duration of vedolizumab treatment, baseline UDCA use and dose, and past use of anti–tumor necrosis factor medication.

Clinical laboratory parameters collected at baseline and follow-up included serum ALP, ALT, AST, bilirubin and albumin, platelet count, and international normalized ratio. ALP was measured in international units per liter and calculated times the ULN. Follow-up laboratory tests were included up until LT, death, or 56 days following the final vedolizumab infusion (because this is the usual duration between maintenance infusions), whichever occurred first.

### Time-Points of Liver Biochemistry Data

Liver biochemistry data (ALP, ALT, AST, and bilirubin levels) were collected at baseline, Week 6 (ie, Day 42), Week 14 (ie, Day 96), and last follow-up while on vedolizumab. The time points of Week 6 and Week 14 were used as early and medium time points to assess change in liver biochemistry because these correspond with the last induction dose of vedolizumab and the first maintenance dose time points. In clinical practice, the response of IBD to vedolizumab treatment is typically assessed after 6–10 weeks.^1^, ^2^

Liver biochemistry results were included up until 56 days after the final vedolizumab infusion, because 56 days is the interval between vedolizumab maintenance doses. For these time point analyses, only patient data that had paired baseline and the appropriate time point data were used. To allow for variation in clinical practice, laboratory test results from Days 30–59 could be assigned as the Week 6 value, and from Days 90–119 were used for the Week 14 value.

### Statistical Analyses

In comparing continuous variables from baseline to a later time point, paired Student *t* tests or Wilcoxon matched-pairs signed rank tests were used according to whether the data were distributed parametrically or nonparametrically, respectively.

Univariate logistic regression was carried out for the outcomes of ALP reduction ≥20% from baseline to last follow-up, ALP rise ≥20% or more from baseline to last follow-up, ALP <1.5 × ULN at last follow-up (within subgroup with baseline ALP >1.5 × ULN), the presence of liver-related outcomes, and improved endoscopic IBD response (vs worsened/unchanged). Multivariate logistic regression was carried out to assess the impact of relevant variables on ALP drop or rise by 20% or more from baseline to last follow-up. Separate univariate and multivariate logistic regression was carried out on ALP drop by 20% or more for the cohort of patients who were not also on UDCA at vedolizumab initiation, because UDCA use can lower ALP. Where there were missing covariates, a complete case analysis was carried out (Supplementary Table 2).

The statistical software used was GraphPad Prism version 7.0a for Mac OS X (GraphPad Software, La Jolla, CA, www.graphpad.com), and for the univariate and multivariate analyses, R 3.5.1 Core Team (R: A language and environment for statistical computing. R Foundation for Statistical Computing, Vienna, Austria, 2018. URL http://www.R-project.org/).

## Supplementary Results

### Duration of Vedolizumab and Follow-up

The median duration of vedolizumab treatment was 412 days (IQR, 180–651; range, 37–2609), and patients were followed up with regards to clinical endpoints for a median duration of 561 days (IQR, 325–790; range, 42–2614). All patients had received a minimum of 3 infusions at the usual dosing schedule, except for 1 patient whose third infusion was slightly earlier on Day 37 rather than Day 42/Week 6. Forty-four patients (43.1%) had their vedolizumab treatment stopped during the study period. Most treatment cessation was caused by lack of efficacy (32 patients; 72.3%), with 6 (13.6%) stopping because of an adverse reaction and another 6 stopping for unknown reasons.

### Timepoints of Last Follow-up Liver Biochemistry Variables

The median duration postvedolizumab of the last follow-up laboratory measurement varied depending on the biochemistry variable. These were: ALP, 374 days (IQR, 167–579); ALT, 374 days (IQR, 166–613); AST, 438 days (IQR, 208–641); and bilirubin, 385 days (IQR, 167–637).

### Outcomes on Subsets of Patients

#### Patients with cirrhosis

Within the cohort of patients with cirrhosis (n = 21) no correlation was found between change in ALP and change in other parameters of liver and organ function, such as international normalized ratio, bilirubin, and creatinine (data not shown).

#### Patients not on ursodeoxycholic acid

Separate univariate and multivariate analyses were carried out on the subgroup of patients who were not on UDCA at baseline (n = 41; Supplementary Table 4). Ten patients (24.4%) had an ALP drop ≥20%; cirrhosis again was associated with this outcome in this cohort on univariate analysis, but not on multivariate analysis.

#### Patients with baseline alkaline phosphatase >1.5 *×* upper limit of normal

Given the previous evidence for a worse prognosis in patients whose ALP is >1.5 × ULN,^20^, ^21^, ^22^ we also evaluated within the subgroup of patients whose baseline ALP was >1.5 × ULN, looking at the outcome of the proportion whose ALP dropped to <1.5 × ULN at last follow-up.

When analyzing this subgroup of patients (ALP >1.5 × ULN at baseline; n = 51) 10 (19.6%) had ALP <1.5 × ULN at last follow-up. Univariate analyses revealed that only type of IBD was associated with this outcome, with an odds ratio of 0.07 (95% confidence interval, 0.013–0.39; *P* = .002) for UC as compared with CD/IBD-unspecified (Supplementary Table 5).

#### Patients with duration of vedolizumab >6 months

Seventy-three patients had been on vedolizumab for a minimum of 6 months and had ALP data to >6 months. We analyzed this subgroup to determine if a longer duration of vedolizumab treatment had any bearing on the previously mentioned results. In this cohort, the median ALP did not change from baseline (1.65 × ULN; IQR, 0.93–3.01) to last follow-up (1.63 × ULN; IQR, 1.03–3.56; *P* = .284), and a similar proportion of patients had an ALP drop ≥20% (24.7%) and ALP rise ≥20% (37.0%) compared with the overall cohort. Univariate and multivariate analyses were also similar to those of the overall cohort (data not shown).
